# Do good psychosocial working conditions prolong working lives? Findings from a prospective study in Sweden

**DOI:** 10.1007/s10433-021-00672-0

**Published:** 2021-12-18

**Authors:** Johanna Stengård, Constanze Leineweber, Marianna Virtanen, Hugo Westerlund, Hui-Xin Wang

**Affiliations:** 1 Stress Research Institute, Department of Psychology, Stockholm University, 106 91 Stockholm, Sweden; 2 School of Educational Sciences and Psychology, University of Eastern Finland, Joensuu, Finland

**Keywords:** Retirement timing, Psychosocial working conditions, Job resources, Prolong working life

## Abstract

Due to an ageing population, governments in European countries are striving to keep older workers longer in the workforce. Remarkably few studies have paid attention to the influence of psychosocial working conditions on timing of retirement for older workers in and beyond normative retirement age. The aim of the present study was to examine whether good psychosocial working conditions contribute to prolonged working lives among older workers (59 years and above). A particular question was whether such conditions increase in importance with age. Seven waves (2006–2018) of the Swedish Longitudinal Occupational Survey of Health (SLOSH) were used (*N* = 6000, observations = 10,632). Discrete-time event history analyses showed that higher levels of job resources (decision authority [OR 1.13, 95% CI 1.06–1.22], skill use [OR 1.17, 95% CI 1.07–1.29], learning opportunities [OR 1.22, 95% CI 1.13–1.31], social support [OR 1.29 (95% CI 1.16–1.42], work-time control [OR 1.07, 95% CI 1.01–1.13], and reward [OR 1.40, 95% CI 1.24–1.57])—but not lower levels of job demands (quantitative and emotional demands or effort)—were associated with working longer (continued work two years later). Also, low effort-reward imbalance (OR 0.84 [95% CI 0.73–0.96]) was associated with working longer. In addition, skill use, work-time control, reward, and low effort-reward imbalance increased in importance with age for continued work. These results suggest that providing older workers with control over their work tasks, giving opportunities for learning and using their skills, as well as rewarding and acknowledging their achievements, may keep them in the workforce longer. Especially, job resources may grow in importance with age.

**Supplementary Information:**

The online version contains supplementary material available at 10.1007/s10433-021-00672-0.

## Introduction

Due to ageing populations, governments in European countries are striving to keep older workers longer in the workforce (Rechel et al. 2013). In fact, according to recent statistics from the European Union, the share of older workers in the workforce has increased during the past 15 years in most European countries. However, despite different reforms of the pensions systems, the number of workers above 65 years of age is still rather low. In 2018, less than 2% of the European population aged 65–69 years were in paid work (Eurostat 2019). To legislate a higher statutory retirement age without other measures might not be a feasible way. In parallel, the sustainability of the work environment should be addressed in order to make it possible and attractive for older workers to work longer. Earlier studies examining the influence of psychosocial working conditions on timing of retirement have been more concerned with early retirement (voluntary or due to disability) rather than late or postponed retirements (cf. Browne et al. 2019). In addition, it is unknown whether the importance of certain psychosocial working conditions may change with age. Accordingly, the aim of the present study was to investigate whether psychosocial working conditions contribute to prolonged working lives among those aged 59 years and above. In particular, whether the importance of psychosocial working conditions increased with age.

The Job Demands–Resources (JD–R) theory (Bakker et al. 2003; Demerouti et al. 2001) is useful to explain a potential link between work environment and prolonged working life. According to the JD–R theory, there are two types of psychosocial working conditions: (1) job demands—characteristics of the job that require persistent effort (e.g. working fast and hard) and thus are associated with costs—and (2) job resources—characteristics of the job that facilitate management of job demands or goal achievements, or embrace personal growth and development (e.g. autonomy and support) (Bakker et al. 2003). Job demands and job resources affect work-related outcomes through two different processes (Bakker et al. 2005). *The health impairment process* postulates that high job demands predict exhaustion (Bakker et al. 2014). This resembles the ‘push’ mechanisms, that is, factors that push older workers out of the labour market, such as high job demands exceeding one’s physical and mental capacity, which may make it too strenuous for some individuals to continue working when ageing (Andersen and Sundstrup 2019). *The motivational process*, on the other hand, postulates that high job resources predict work engagement (Bakker et al. 2014). This resembles ‘stay’ (also called ‘pull’) mechanisms, that is, factors that attract older workers to voluntarily continue working (Andersen and Sundstrup 2019). According to the JD–R theory, job resources may also buffer the impact of high job demands on exhaustion (Bakker et al. 2014). Here especially job strain (cf. DCM model by Karasek 1979; Karasek and Theorell 1990), defined by combinations of (quantitative) psychological demands and job control (i.e. passive, low-strain, active, or high-strain jobs), has frequently been studied in relation to health. Another prominent model related to demands and resources is the effort–reward imbalance (ERI) model (Siegrist 1996), which claims that an imbalance between high efforts spent at work and low reward received in turn (money, esteem, career opportunities, job security) has negative consequences for worker’s health.

Since poor health is known to affect retirement age, there is reason to believe that both low job demands and high job resources and beneficial combinations thereof—through their influence on health, work ability, and motivation—increase the likelihood of working longer among workers of pensionable age. Also, there are reasons to believe that psychosocial working conditions may have a stronger impact on the decision to continue working versus retiring when individuals approach and reach the normative retirement age, as there are both less normative requirements and economic necessity to continue working, whereas retirement can become an increasingly attractive option expected by society. For example, high job demands may be tougher to keep up with as working capacity and health deteriorates with age (Götz et al. 2018), and the importance of job resources may increase because they not only need to compensate for, but exceed the benefits that are linked to retirement. Therefore, we hypothesise that the importance of psychosocial working conditions on continued work increases with age.

The statutory pension age varies between European countries (Eurostat 2019). In Sweden, although the Swedish pension system is rather flexible with no fixed statutory retirement age, still 65 years is the normative retirement age (Anxo et al. 2017; SOU 2020: 69). During the data collection of this study, from the age of 61^1^ (for both men and women) it was possible to retire (part- or full-time) and start receiving collected earnings-related state and civil servant^2^ pension, while guarantee pension, paid to individuals with low collected earnings, was paid from 65. The employee had a legal right to continue working until the 67th birthday,^3^ after which the individual could continue to work if agreed with the employer (https://www.pensionsmyndigheten.se).

### Empirical studies on psychosocial working conditions and timing of retirement

With regard to *early* retirement, job demands and job resources are well-studied. According to a systematic review (Browne et al. 2019), high job demands were associated with intention to retire later, but not consistently with the timing of actual retirement. The evidence for a negative association between job resources, such as decision latitude and social support, and early retirement are more solid (Browne et al. 2019). Associations between job strain or ERI and actual early exit were not supported in one study (Robroek et al. 2013), but have been found between ERI and early retirement intentions in two other studies (Siegrist et al. 2007; Wahrendorf et al. 2013).

However, studies focusing on retirement timing not linked to early retirement or disability pension are still scarce. Some exceptions are a few studies that did not find support for a link between job demands and working longer (Carr et al. 2016; Van Solinge and Henkens 2014), whereas in terms of job resources, it was found that decision authority (Carr et al. 2016) or decision authority in combination with good health (Jonsson et al. 2019) increased the odds for working longer. Moreover, growth and promotion opportunities, recognition, and social support at work were also associated with prolonged working lives, whereas job challenge, training opportunities, and flexibility of work-time and place were not (Carr et al. 2016; Van Solinge and Henkens 2014). To date, neither job strain or ERI have received much attention with regard to prolonged working life (Browne et al. 2019) and associations between job strain (Carr et al. 2016; Virtanen et al. 2014) or ERI (Virtanen et al. 2014) and working longer have not been supported.

Finally, a limited number of studies have focused especially on working conditions that could influence individuals to work beyond statutory retirement age by distinguishing individuals that stopped working before statutory retirement age from those who continued working after statutory retirement age. These studies show mixed results. For example, one Danish study found that low quantitative demands were associated with working beyond statutory retirement age (65 years) (Andersen et al. 2021), whereas no such associations were supported in a series of studies on a sample of older workers from the Netherlands focussing on *bridge employment* (combination of having pension while working after 65 years) versus full retirement at 65 years or earlier (de Wind et al. 2016; van der Zwaan et al. 2019). With regard to job resources, these latter studies found that workers who felt appreciated by colleagues and supervisors retired later (de Wind et al. 2016; van der Zwaan et al. 2019), but no associations between working beyond retirement age and decision authority, social support, or learning opportunities, respectively, were found (de Wind et al. 2016). The Danish study supported a link between several job resources (e.g. decision authority, recognition from management, and possibility for development) and working beyond retirement age (Andersen et al. 2021). Yet another study found that older retirees (more than six month after the statutory retirement date) reported more work-time control before retiring than younger retirees (Virtanen et al. 2014). Apart from the fact that these latter studies only distinguish between two groups, the exposures were often measured rather long time before the statutory retirement age, which means that for late pensioners, changes in working conditions preceding the years of retirement are not taken into account. Moreover, to our knowledge, no studies have examined whether the importance of psychosocial working conditions on actual retirement increases with age.

### Hypotheses

Based on the review above, we hypothesise that: working longer (continued work in any of a 2-year follow-up intervals) among older workers (59 years and older) is predicted by lower levels of job demands (quantitative demands, emotional demands, and effort) (H1), higher levels of job resources (decision authority, skill use, learning, social support, work-time control, and reward) (H2), active, passive, and low-strain jobs compared to high-strain jobs (H3), and lower levels of ERI (H4). Additionally, we hypothesise that the importance of psychosocial working conditions for continued work increases with age (H5).

## Methods

### Sample and procedure

The Swedish Longitudinal Occupational Survey of Health (SLOSH) is a national cohort study, biennially collected since 2006. There are two versions of the SLOSH questionnaire: ‘*work questionnaire*’ (for those working 30% of full-time or more) and ‘*non-work questionnaire*’ (for those working less than 30% of full-time or not working at all). The respondents are requested to fill in the one that corresponds best to their situations. The SLOSH cohort is built upon a nationwide, representative sample of the Swedish working population, originally commissioned by the Swedish Work Environment Authority for collecting repeated cross-sectional surveys of the Swedish working force, the Swedish Work Environment Survey (SWES). At the SLOSH baseline (2006), the SWES respondents from 2003 were invited to participate in SLOSH. Eventually, more cross-sectional SWES populations have been added and from 2014, SLOSH comprised of all SWES participants 2003–2011 (≈40,000 individuals). A detailed description of SLOSH can be found elsewhere (Magnusson Hanson et al. 2018). The present study was approved by the Regional Research Ethics Board in Stockholm.

#### Inclusion criteria

The present study included participants from SLOSH 2006–2018. Individuals who responded to the ‘work questionnaire’ at any of the six first waves and at that time had reached at least the age of 59 (i.e. reaching the lowest age for old age pension of 61 years at the follow-up, two years later) were eligible for the study (*n* = 7900). In the present study, an observation consists of a pair of waves, i.e. ‘baseline wave’ (any of the six first waves) and ‘follow-up wave’ (the subsequent wave). Observations were selected if the individual at the baseline wave answered the ‘work questionnaire’ and at the follow-up wave answered either the ‘work questionnaire’ or the ‘non-work questionnaire’ and indicated that the reason for not working anymore was full-time old age retirement. Observations where the person at either the baseline or the follow-up wave still worked, but worked less than 30% (for instance due to part-time retirement) were not taken into account. The final dataset (*n* = 6000; 10,632 observations) included observations from those individuals who had completed at least one work–work or work–retirement transition between two subsequent waves. This means that each person could contribute with one to six observations. A few individuals were re-employed after their first retirement (*n* = 95) and only contributed to the analyses with their first work–retirement transition and any previous work–work transitions.

### Measures

#### Outcome variable

*Working longer* (continued work (1) vs. retired (0)) was measured at follow-up (two years later). Individuals were regarded as still working if they answered the ‘work questionnaire’ (i.e. working at least 30% of a full-time) and retired if they answered the ‘non-work questionnaire’ and there stated being full-time retired with old age pension (excluding disability retirement).

#### Exposure variables

Psychological working conditions were assessed with three job demands scales and six job resources scales. All items were measured on Likert scales with 4-degree responses, except work-time control which had five response alternatives. The Swedish versions of all scales have previously been validated. Mean indices were estimated for individuals who had answered more than 50% of the items of a particular scale. The scales were reversed (when necessary), so that higher values represent higher levels of demands and resources. Table 1 shows descriptives for the exposure variables and their Cronbach’s alphas.

**Table 1:** Descriptive statistics for exposure variables and Cronbach’s alpha

	Value range	Waves measured	No. of observations	Cronbach’s alpha	Mean (SD)
Quantitative demands	1: “no, almost never” to 4: “yes, often”	T1–T7	10,524	0.74	2.53 (.55)
Emotional demands*	1: “no, almost never” to 4: “yes, often”	T1–T7	10,515	–	2.67 (.92)
Effort	1: “don’t agree” to 4: “agree completely”	T3–T7	8366	0.79	2.53 (.74)
Decision authority	1: “no, almost never” to 4: “yes, often”	T1–T7	10,474	0.75	3.20 (.74)
Skill use*	1: “no, almost never” to 4: “yes, often”	T1–T7	10,520	–	3.66 (.54)
Learning opportunities*	1: “no, almost never” to 4: “yes, often”	T1–T7	10,516	–	3.17 (.68)
Social support	1: “no, almost never” to 4: “yes, often”	T1–T7	10,259	0.86	3.22 (.52)
Work-time control	1: “very little” to 5: “a high degree”	T2–T7	9694	0.89	2.92 (1.09)
Reward	1: “don’t agree” to 4: “agree completely”	T3–T7	8306	0.69	2.65 (.50)
ERI	.25–4.00	T3–T7	8300	–	1.02 (.44)

^*^Single item

#### Job demands

*Quantitative demands* were assessed by a 5-item-scale (working fast, working intensively, too much effort, (not) enough time, and conflicting demands) from the Demand–Control–Support-Questionnaire (DCSQ) (Chungkham et al. 2013; Theorell et al. 1988). *Emotional demands* were measured with one item (“Does your work place you into emotionally disturbing situations?”) from the Copenhagen Psychosocial Questionnaire (COPSOQ) (Pejtersen et al. 2010). *Effort*^4^ was measured by a 3-item-scale (Li et al. 2019) from the ERI model, which deals with interruptions and disturbances, increased workload, and time pressure due to heavy workload.

#### Job resources

*Decision authority*, one of the dimensions of decision latitude (i.e. control), was measured with two items (what to do and how to do the work) from the DCSQ (Chungkham et al. 2013; Theorell et al. 1988). Both *skill use* (“Does your work demand a high level of skill or expertise?”) and *learning opportunities* (“Do you have the possibility of learning new things through your work?”) were measured with single items derived from the DCSQ subscale skill discretion. *Social support* was assessed by a 6-item-scale from the DCSQ concerning atmosphere, understanding and cohesion among colleagues and managers. *Work-time control*^5^ was measured by a 6-item-scale (Ala-Mursula et al. 2002), which assesses the opportunity to influence one’s working time (start and end times, length of working day, taking breaks, running private errands during work-time, which days to work, and holidays). *Reward*^6^ was measured by a 7-item-scale (Li et al. 2019) from the ERI model, which concerns job promotion (adequate salary, work and promotion prospects), job security (job security and not experience/expecting undesirable changes) and esteem (receive the deserved acknowledgement, respect and prestige).

#### Job demands and resources combined

*Job strain*, the combination of demands and control, was here measured with a variable combining the binary variables (with the medians as cut-off) for quantitative demands and decision authority^7^: *high-strain* (high demands, low control), *active* (high demands, high control), *passive* (low demands, low control), and *low-strain* (low demands, high control) (Karasek 1979). *ERI ratio* is calculated based on a predefined algorithm (score effort/score reward, adjusted for unequal number of items) (Li et al. 2019), where a high value corresponds to an imbalance between effort and reward, such that the reward fall short of the effort.

#### Covariates

Age, gender, occupational position, income, and family situation are variables that are commonly adjusted for in retirement studies (Fisher et al. 2016; Sousa-Ribeiro et al. 2021), as they may act as potential confounders in the association between psychosocial working conditions and retirement timing. For example, those in blue-collar occupations generally have worse working conditions and tend to retire earlier (Carr et al. 2018), whereas self-employed persons often report higher work control (Hessels et al. 2017) and work longer (SOU 2020: 69). We also, in compliance with other studies (see e.g. Thorsen et al. 2016; Virtanen et al. 2014), controlled for part-time and shift work—two variables tied to employment conditions—that may associate with both psychosocial working conditions and retirement timing. Information on age, gender, and income (logarithmic value) were register-based. Occupational position (blue-collar, white-collar, or self-employed) was based on SEI (Statistics Sweden 1984)—a categorisation based on occupation and education level provided by Statistics Sweden—and a question regarding self-employment. Individuals were categorised as self-employed according to the SEI or self-report. Working time (working full- vs part-time), marital status (being married/cohabitant vs single), parental status (having children living at home or not), shift work (daytime vs other work-time) were self-reported. To adjust for potential timing effects, also wave (categorical) was included as a covariate.

### Statistical analysis

For the study purpose, we employed discrete-time event history analysis models with the conditional probability of the binary outcome working longer (continued work vs. retired) measured at follow-up. The statistical analyses were run with Stata version 16.1. This was accomplished by performing logistic regressions with clustering of observations over the same individual, meaning that standard errors are adjusted with a robust cluster variance estimator. Our data admit one to six transitions (observations) between two successive waves (from work to work or from work to retirement) for each individual, and the exposures and covariates were all measured at each baseline wave. In the models, age (based on registry data and corresponding to the person’s age at the baseline wave of each particular observation) served as the timing of the event variable. Because the association between age and the outcome variable *continued work *(*vs. retired*) was U-shaped (see Supplementary material), age was represented in all models by a linear and a quadratic term.^8^ Besides age, in all models, gender and wave were included as covariates (Model 1). Occupational position, working time, marital status, parental status, shift work, and income were included in the fully adjusted models (Model 2).

We tested whether there was an interactive effect between linear age and demand/resource on working longer by adding an interaction term between (linear) age and the demand/resource (Model 3) to the fully adjusted model. By comparing models with Wald *χ*^2^ test, we examined whether the added interaction term gave any significant contribution.

Sensitivity analyses were performed: (1) age variable was entered as a categorical variable instead of as linear and quadratic terms (in Model 1–2), and (2) the oldest workers were excluded (70 and above) as corresponding to few observations (in Model 1–3). Both provided very similar results (not shown) to the reported results. Exception was the interaction term between age and learning opportunities in Model 3 (sensitivity analysis 2) which turned statistically significant.

## Results

At the initial survey (time point varies between the 6000 participants), 54.4% were women, 29.7% blue-collar workers, 61.3% white-collar workers, and 9.0% self-employed. Moreover, 75.3% worked full-time and 24.7% worked part-time (at least 30% of a full-time), 79.1% were married/cohabiting, and 11.2% had children living at home. Table 1 provides descriptive statistics for the exposure variables, and Table 2 shows the distribution of observations per age by working longer (continued work vs retired two years later).

**Table 2:** Distribution of observations over continued work or retired two years later by age (at baseline, before transition)

Age at baseline	Age at follow-up	Retired at follow-up	Working at follow-up	Total observations
59	61	80 (4.7%)	1635 (95.3%)	1715
60	62	137 (7.9%)	1605 (92.1%)	1742
61	63	258 (15.1%)	1451 (84.9%)	1709
62	64	405 (26.0%)	1154 (74.0%)	1559
63	65	847 (59.4%)	580 (40.6%)	1427
64	66	794 (66.2%)	406 (33.8%)	1200
65	67	339 (65.2%)	181 (34.8%)	520
66	68	236 (64.8%)	128 (35.2%)	364
67	69	61 (38.6%)	97 (61.4%)	158
68	70	44 (44.9%)	54 (55.1%)	98
69	71	26 (41.3%)	37 (58.7%)	63
70–76	72–78	20 (26.0%)	57 (74.0%)	77
Total		3247 (30.5%)	7385 (69.5%)	10,632

The number of observations differed slightly in the analyses depending on response rate of a particular psychosocial work environment factor

### Main contribution of psychosocial working conditions on working longer

Table 3 shows that neither quantitative or emotional demands nor effort were significantly associated with working longer. Similar results were observed in the minimally adjusted Model 1 (adjusted for age, wave, and gender) and fully adjusted Model 2 (additionally adjusted for occupational position, income, working time, shift-time, marital status, and parental status). All job resources, in turn, were associated with higher odds for working longer both in models minimally and fully adjusted for the covariates. In the fully adjusted model, higher ORs of working longer were related to one unit increase in the decision authority scale (OR 1.13, 95% CI 1.06–1.22), skill use (OR 1.17, 95% CI 1.07–1.29), learning opportunities (OR 1.22, 95% CI 1.13–1.31), social support OR 1.29 (95% CI 1.16–1.42), work-time control (OR 1.07, 95% CI 1.01–1.13), and reward (OR 1.40, 95% CI 1.24–1.57), respectively.

**Table 3:** Odds ratio (OR) and 95% confidence interval (CI) of working longer [continued work (1) vs. retired (0) two years later] in relation to job demands and resources (one unit increase on the scale); separate models

	Model 1. Minimally adjusted	No. of observations (no. of clusters/individuals)	Model 2. Fully adjusted	No. of observations (no. of clusters/individuals)
*Job demands*				
Quantitative demands	0.94 (.86–1.03)	10,524 (5965)	0.93 (.85–1.03)	9677 (5680)
Emotional demands	1.04 (.98–1.10)	10,515 (5967)	1.05 (.99–1.11)	9667 (5678)
Effort	1.02 (.94–1.09)	8366 (5115)	1.00 (.93–1.09)	7741 (4880)
*Job resources*				
Decision authority	1.24 (1.16–1.32)*	10,474 (5951)	1.13 (1.06–1.22)*	9617 (5660)
Skill use	1.28 (1.18–1.40)*	10,520 (5966)	1.17 (1.07–1.29)*	9674 (5681)
Learning opportunities	1.30 (1.21–1.39)*	10,516 (5964)	1.22 (1.13–1.31)*	9668 (5676)
Social support	1.31 (1.19–1.44)*	10,259 (5864)	1.29 (1.16–1.42)*	9474 (5591)
Work-time control	1.16 (1.11–1.22)*	9694 (5643)	1.07 (1.01–1.13)*	8967 (5381)
Reward	1.44 (1.29–1.61)*	8306 (5085)	1.40 (1.24–1.57)*	7699 (4854)

Model 1. Adjusted for age (linear & quadratic: where 59 years = 1), gender, wave

Model 2. Same adjustments as Model 1 plus occupational status, marital status, parental status, working time, shift work, and income

All job demands and resources measured on scale 1–4, except WTC (1–5)

*For *p* < .05; ^†^For .05  ≤ *p* < .10

With regard to combinations of job demands and resources (Table 4), compared to *high-strain* jobs (high quantitative demands and low decision authority), both *active* jobs (high quantitative demands and high decision authority, OR 1.27 [95% CI 1.10–1.46]) and *low-strain* jobs (low quantitative demands and high decision authority, OR 1.21 [95% CI 1.05–1.39]), but not *passive* jobs were associated with higher odds for working longer in both the minimally and fully adjusted models. Finally, ERI was associated with lower odds for working longer both in the minimally and the fully adjusted model (OR 0.84 [95% CI 0.73–0.96]).

**Table 4:** Odds ratio (OR) and 95% confidence interval (CI) of working longer [continued work (1) vs. retired (0) two years later] in relation to job strain and Effort-Reward imbalance (ERI) (one unit increase on the scale); separate models

	Model 1. Minimally adjusted	No. of observations (no. of individuals)	Model 2. Fully adjusted	No. of observations (no. of individuals)
Job strain categories^a^				9606 (5654)
*high-strain (H;L)*	1	(*n* = 2906)	1	(*n* = 2690)
*active (H;H)*	1.42 (1.24–1.62)*	(*n* = 2601)	1.27 (1.10–1.46)*	(*n* = 2384)
*passive (L;L)*	1.01 (.88–1.16)	(*n* = 2127)	1.04 (.90–1.21)	(*n* = 1968)
*low-strain (L;H)*	1.36 (1.19–1.55)*	(*n* = 2808)	1.21 (1.05–1.39)*	(*n* = 2564)
Effort-reward imbalance				
One unit increase^b^	0.84 (.74–.95)*	8300 (5083)	0.84 (.73–.96)*	7693 (4852)

Model 1. Adjusted for age (linear and quadratic: where 59 years = 1), gender, wave

Model 2. Same adjustments as Model 1 plus occupational status, marital status, parental status, working time, shift work, and income

^a^Quantitative demands (Low or High); decision authority (Low or High). High and low values are divided according to median of the scale (quantitative demands = 2.60; decision authority = 3.50)

^b^Value range 0.25–4.0

*For *p* < .05; ^†^For .05  ≤ *p* < .10

### Linear influence of age on the association between psychosocial working condition and working longer

**Fig. 1:**
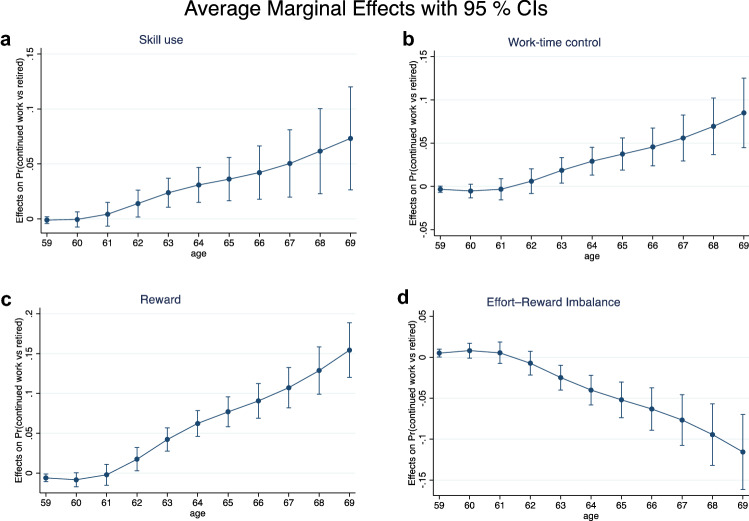
Average marginal effects of one SD increase in **a** skill use, **b** work-time control, **c** reward, and **d** effort-reward imbalance on the likelihood of working longer (continued work vs retired at 2-year follow-up) by age, up to age 69. *Note* the graphs present different scales on y-axis

Table 5 (Model 3) shows that there was no linear effect of age on the association between job demands and working longer (continued work vs retired at 2-year follow-up). For job resources, significant interaction terms indicated that skill use (Wald = 4.94 [*p* < 0.05]; OR 1.07 [95% CI 1.01–1.13]), work-time control (Wald = 10.48 [*p* < 0.01]; OR 1.05 [95% CI 1.02–1.08]), and reward (Wald = 30.28 [*p* < 0.001]; OR 1.21 [95% CI 1.13–1.30]) increased in importance with age for working longer. Figure 1 presents the average marginal effects (AME) of one standard deviation (SD) increase in the job resources score on the likelihood of working longer (continued work vs retired at 2-year follow-up) for different ages, i.e. the differences in predicted proportion of people still working in case of one SD increase in the job resources score (Williams 2012). We restricted the diagrams to ages up to 69 years since the observations for older ages were too few to make reliable predictions. For example, Fig. 1a shows that with regard to skill use, an AME 0.07 for those aged 69, which means that the predicted proportion of people still working in next two years is seven percentage higher for one SD above the mean of the skill use score than those who have the mean value, and the corresponding figure for those aged 64 years was 0.03. This indicates that the importance of skill use for continued work increased with age, so that, for example, one SD increase in skill was associated with a twofold probability of continued working among people who still worked at age 69 when compared with 64-year-old.

**Table 5:** Linear influence of age on the association between demand/resource and working longer [continued work (1) vs. retired (0) two years later]

	Model 3. Fully adjusted		
	Main/Linear interaction	OR (95% CI)	Wald *χ*^2^-test (*p*-value)
*Job demands*			
Quantitative demands	Main effect	0.93 (.72–1.22)	
	Interaction with age	1.00 (.95–1.06)	0.00 (.991)
Emotional demands	Main effect	1.06 (.91–1.24)	
	Interaction with age	1.00 (.97–1.03)	0.02 (.884)
Effort	Main effect	1.23 (.98–1.54)†	
	Interaction with age	0.96 (.92–1.00)†	3.52 (.061)†
*Job resources*			
Decision authority	Main effect	1.16 (.95–1.40)	
	Interaction with age	1.00 (.96–1.03)	0.06 (.809)
Skill use	Main effect	0.86 (.65–1.15)	
	Interaction with age	1.07 (1.01–1.13)*	4.94*
Learning opportunities	Main effect	1.07 (.85–1.33)	
	Interaction with age	1.03 (.98–1.08)	1.53 (.216)
Social support	Main effect	1.05 (.78–1.41)	
	Interaction with age	1.04 (.98–1.11)	2.03 (.154)
Work-time control	Main effect	0.85 (.74–.99)*	
	Interaction with age	1.05 (1.02–1.08)**	10.48**
Reward	Main effect	0.55 (.38–.78)***	
	Interaction with age	1.21 (1.13–1.30)***	30.28***
ERI	Main effect	1.76 (1.16– 2.68)**	
	Interaction with age	0.85 (.78–.93)***	14.01***

Model 3. Fully adjusted for gender, wave, occupational status, marital status, parental status, working time, shift work, and income

Main effect of age (linear term and for quadratic term) not shown

***For *p* < .001; **For *p* < .01; *For *p* < .05; ^†^For .05  ≤   *p* < .10

Similar patterns could be found for work-time control (Fig. 1b) and reward (Fig. 1c). Also, there was a significant interaction effect between age and ERI on continued work (Wald = 14.01 [*p* < 0.001]; OR 0.85 [95% CI 0.78–0.93]), where higher ERI decreased the likelihood of continued work with increasing age at least up to 69 years (Fig. 1d).

## Discussion

In the present study, we examined whether good psychosocial working conditions, in terms of low job demands (quantitative demands, emotional demands, and effort) and high job resources (decision authority, skill use, learning opportunities, social support, work-time control, and reward) contributed to working longer among older employees and whether the strengths of these associations increased with age. Utilising data from participants 59 years and above drawn from an approximative representative cohort of the Swedish working population, we found support for associations between job resources—but not job demands—and continued work. The strengths of the associations between certain job resources and working longer increased with age.

Hypothesis 1 was not supported as associations between job demands and working longer were not found, which is in line with the majority of previous studies showing that job demands alone, such as quantitative and emotional demands, were not associated with retirement (Browne et al. 2019; Carr et al. 2016; Van Solinge and Henkens 2014). Moreover, age had no influence on the associations between job demands and continued work, which was a bit surprising as we hypothesised that ageing would make it harder for some individuals to cope with high job demands, and thus would act as push factors into retirement in accordance with *the health impairment process* of the JD–R theory stating that high job demands predict exhaustion (Bakker et al. 2014). However, individuals with poor mental and physical capacity may before reaching the retirement age already have left the labour market due to, for instance, disability pension or long-term sickness absence.

With regard to job resources, in line with earlier studies examining psychosocial working conditions on actual retirement age (Andersen et al. 2021; Carr et al. 2016; Virtanen et al. 2014), we found that decision authority, skill use, learning opportunities, social support, work-time control, and reward were positively associated with working longer. Thus, hypothesis 2 was supported. This finding is in line with *the motivational process* of the JD–R theory (Bakker et al. 2014), stating that high job resources predict work engagement. Moreover, using one’s skills, having opportunities to learn, perceiving control over one’s work tasks and time and receiving social support pertain to the three fundamental psychological needs: competence, autonomy and relatedness, of the self-determination theory (Ryan and Deci 2000). Job resources, thus, attract older workers to voluntarily stay in the labour market (Andersen and Sundstrup 2019).

With regard to job strain—the combination of quantitative demands and decision authority—hypothesis 3 was partly supported because workers in both active and low-strain jobs were more inclined to work longer compared to workers in high-strain jobs. This finding is not in line with the scarce existing evidence, which does not support a link between job strain categories and working longer (Carr et al. 2016; Robroek et al. 2013; Virtanen et al. 2014). However, two of these studies utilised different categorisations of job strain compared to ours, that is, Virtanen et al. (2014) calculated the difference between job demands and job control, a strategy that fails to differentiate between active and passive jobs, and Robroek et al. (2013) utilised the effort scale as the job demand variable and studied only early retirement. To be noted, in sensitivity analyses no interaction was found between quantitative demands and decision authority on the likelihood of working longer, which indicates that it was primarily decision authority (and not quantitative demands) that accounted for the influence on working longer. With regard to imbalance between effort and reward, in contrast to the few previous studies (Robroek et al. 2013; Virtanen et al. 2014), we did find support for an overall association between lower ERI and working longer, supporting hypothesis 4.

To our knowledge, the present study is the first which examined the impact of age on the association between psychosocial working conditions and timing of retirement. Partly supporting hypothesis 5, our findings suggest that skill use, work-time control, reward, and learning opportunities, as well as when the balance between effort and reward is favourable may increase in importance with age (at least up to 69) for continued work. One reason for this may be that job resources need to compensate in the form of work engagement for deferred pension benefits, such as time for leisure and other valuable things in life (SOU 2020: 69), and that working beyond normative retirement age may conflict with societal norms (Anxo et al. 2017). For example, more flexible work-time arrangements may facilitate work at older ages. However, conclusions about the older age groups must be drawn with some caution as the number of observations approaching 70 and above in our study was relatively few. This is expected because the majority of workers have retired by then. Nevertheless, more research is warranted on this older age category.

The associations presented in this study may underestimate the importance of resources for the motivation to work longer since the timing of retirement in many cases is involuntary, due to forced earlier (e.g. job loss, health limitations, family reasons) or later (e.g. financial necessity) retirement (Solem et al. 2016; Steiber and Kohli 2017) and strong social norms (Anxo et al. 2017). Meaning that if the retirement decision was completely up to the individuals’ own preferences, the psychosocial working conditions may have had even stronger implications. Although we adjusted the models for income and family situation, we still cannot completely rule them out as potential confounders, as these factors can impact retirement timing in a more complex way. Also, the role of health on the associations between psychosocial working conditions and retirement timing is complex, where health besides a direct effect on retirement timing, plausibly could act indirectly as a mediator or a moderator on the associations (between psychosocial working conditions and working longer) (Nilsson 2020). For example, systematic reviews show that poor psychosocial working conditions negatively influence health (e.g. Li et al. 2021; Theorell et al. 2015). Thus, including health as a covariate may underestimate the strength of the studied associations, and therefore, we decided against it. However, sensitivity analyses where self-rated health was included as an additional covariate to the fully adjusted models resulted in very similar effect sizes and significance levels as our original analyses (see Supplementary material). For overall effects, only one result differed—ERI turned non-significant (although over time it still increased in importance), suggesting that perhaps self-rated health mediate the influence of ERI on retirement timing. With regard to interactions with age, the significance levels were changed for effort and skill use, although the effect sizes were almost identical. Also, we tested whether there were any interaction effects between self-rated health and psychosocial working conditions in predicting continued work, such that people with poor health in combination with poor working conditions would be less likely to continue their work. However, no such effect was found.

### Strengths and limitations

Strengths of this study are that we tested our hypotheses in a sizeable, approximately representative cohort with many time points and used several well-tested multi-item scales. But as with all research, there are also possible limitations. First, the data collection was originally not tailored for our purpose of study retirement, and therefore, we do not know the exact date of retiring. For example, for each transition, the baseline age of the respondents is known—when exposures are measured—but for individuals retired before the next wave, two years later, we do not know the exact age when they retired. This means that age, although mutually exclusive in terms of age when reporting on work environment, are not completely mutually exclusive in terms of age of retirement. Possibly this might have blurred the age differences, which might have been more distinct with more frequent data collections or perhaps with register data on annual sources (pension vs labour) of income (Jonsson et al. 2019). Despite this possible drawback, we found a tendency towards an increased influence of the work environment on continued work versus retirement in older ages. Second, this study probably includes healthier individuals than average because individuals with the poorest health and working conditions tend not to participate in studies, which can be a particular problem for longitudinal cohort studies. Third, we did not consider observations where the person worked less than 30% of a full-time job, which means that the results does not reveal the significance of working conditions for reducing work-time from over 30% to less than 30%, and from there to retirement. It should also be noted that some exposure scales were introduced in later waves, meaning that the different analyses are based on different number of observations. In spite of these possible limitations, this study contributes with new insights of the influence of job demands and job resources on prolonged working lives and their importance in relation to age.

## Conclusion

Due to ageing populations in Europe, people need to work longer than traditional retirement ages. The present study suggests that job resources are important determinants of prolonged working lives. Moreover, job resources and a good balance between effort and rewards may even grow in importance for continued work during pensionable ages. Thus, it is important that legislators and organisations give their older workers control over their work tasks and work-time, opportunities for learning and using their skills, as well as rewarding and acknowledging their achievements, in terms of salary, work prospects and job security. Also, workers themselves could seek to improve their working conditions by striving for more job resources. More research is warranted on changes in the importance of working conditions with age.

## Supplementary Information

Below is the link to the electronic supplementary material.

Supplementary file1 (DOCX 34 kb)

## Data Availability

Given restrictions from the ethical review board and considering that sensitive personal data are handled, it is not possible to make the data freely available. Access to the data may be provided to other researchers in line with Swedish law and after consultation with the Stockholm University legal department. Requests for data, stored at the Stress Research Institute, Department of Psychology, should be sent to registrator@su.se with reference to “Do good psychosocial working conditions prolong working lives? Findings from a prospective study in Sweden” or directly to the corresponding author.

## Footnotes

1 From 2020, changed to 62 years.

2 The majority of the Swedish employees receive civil servant pension, commonly determined in collective agreements between employers or employers' organisations and trade unions.

3 From 2020, changed to 68 years.

4 The effort scale was included in SLOSH from Time 3 onwards.

5 Work-time control was included in SLOSH from Time 2 onwards.

6 The reward scale was included in SLOSH from Time 3 onwards.

7 Decision authority is one of two dimensions that usually comprises control. The second variable *skill discretion* did not show adequate reliability.

8 Age was modified so that value 1 represented 59 years.
