# Supplementary material for: Do good psychosocial working conditions prolong working lives? Findings from a prospective study in Sweden
Source: Eur J Ageing. 2021 Dec 18;19(3):677–88. doi: 10.1007/s10433-021-00672-0 (PMC9424473; doi:10.1007/s10433-021-00672-0)
Supplement: Supplementary file 1 — Supplementary file1 (DOCX 34 kb) [file 10433_2021_672_MOESM1_ESM.docx]

Supplementary material

Figure S1 shows the odds ratios for working longer. Note that the inversed odds ratios values are shown, that is retired vs continued work at follow-up. As can be noticed, age did not have a linear association with retiring.

Figure S1. Inversed odds ratios for working longer (continued work (1) or retired (0) two years later) as a function of age (categorical), where the reference is 59 years.

Table S1. Odds ratio (OR) and 95% confidence interval (CI) of working longer (continued work (1) vs retired (0) two years later) in relation to job demands and resources (one unit increase on the scale); separate models. Model 2S builds on Model 2. Self-rated health included as a covariate.

|  | **Model 2S**  **Fully adjusted + SRH** | **No. of observations**  **(no. of clusters/**  **individuals)** |
| --- | --- | --- |
| **Job demands** |  |  |
| Quantitative demands | 0.97 (.88–1.07) | 9,597 (5,652) |
| Emotional demands | 1.06 (.998–1.12) | 9,587 (5,650) |
| Effort | 1.04 (.96–1.13) | 7,675 (4,856) |
| **Job resources** |  |  |
| Decision authority | **1.11 (1.03–1.19)*** | 9,537 (5,631) |
| Skill use | **1.16 (1.06–1.28)*** | 9,593 (5,652) |
| Learning opportunities | **1.21 (1.12–1.31)*** | 9,588 (5,648) |
| Social support | **1.23 (1.11–1.36)*** | 9,395 (5,563) |
| Work-time control | **1.06 (1.00–1.12)*** | 8,891 (5,353) |
| Reward | **1.34 (1.18–1.51)*** | 7,633 (4,830) |
|  |  |  |
| **Effort-Reward imbalance** |  |  |
| One unit increase | 0.90 (.78–1.03) | 7,627 (4,828) |
|  |  |  |
| **Job strain categories** ^a^ |  | 9,526 (5,625) |
| *high-strain (H;L)* | 1 | (n=2,660) |
| *active (H;H)* | **1.22 (1.06–1.41)*** | (n=2,371) |
| *passive (L;L)* | 0.99 (.85–1.14) | (n=1,955) |
| *low-strain (L;H)* | 1.15 (1.00–1.33) † | (n=2,540) |

Model 2S. Fully adjusted plus Self-rated health.

^a^ quantitative demands (Low or High); decision authority (Low or High). High and low values are divided according to median of the scale (quantitative demands = 2.60; decision authority = 3.50).

* for *p* < .05; † for .05 < *p* <.10

Table S2. Linear interaction effect between age and demand/resource on continued work.

Model 3S builds on Model 3. Self-rated health included as a covariate.

|  | **Model 3S.**  **Fully adjusted + SRH**  **Adding linear interaction term *(age * demand/resource)*** | | |
| --- | --- | --- | --- |
|  | **Main/**  **Linear interaction** | **OR (95% CI)** | **Wald χ^2^−test**  **(*p*-value)** |
| **Job demands** |  |  |  |
| Quantitative demands | main effect | 0.98 (.75–1.23) | 0.00 (.955) |
|  | interaction with age | 1.00 (.95–1.05) |  |
| Emotional demands | main effect | 1.08 (.92–1.26) | 0.04 (.839) |
|  | interaction with age | 1.00 (.97–1.03) |  |
| Effort | main effect | **1.31 (1.05­–1.64)*** | **4.58*** |
|  | interaction with age | **0.95 (.91–1.00)*** |  |
| **Job resources** |  |  |  |
| Decision authority | main effect | 1.11 (.92–1.35) | 0.00 (.978) |
|  | interaction with age | 1.00 (.96–1.04) |  |
| Skill use | main effect | 0.89 (.67–1.18) | 3.78† |
|  | interaction with age | 1.06 (1.00–1.12)† |  |
| Learning opportunities | main effect | 1.07 (.85–1.34) | 1.31 (.253) |
|  | interaction with age | 1.03 (.98–1.07) |  |
| Social support | main effect | 0.99 (.74–1.34) | 2.21 (.137) |
|  | interaction with age | 1.05 (.99– 1.11) |  |
| Work-time control | main effect | **0.84 (.72–.97)*** | **11.49***** |
|  | interaction with age | **1.05 (1.02–1.08)***** |  |
| Reward | main effect | **0.52 (.36–.74)***** | **29.94***** |
|  | interaction with age | **1.21 (1.13–1.30)***** |  |
| ERI | main effect | **1.97 (1.29–3.02)**** | **15.08***** |
|  | interaction with age | **0.84 (.78–.92)***** |  |

Model 3S. Fully adjusted plus Self-rated health.

*** for *p* <.001; ** for *p* <.01; * for *p* < .05; † for .05 < *p* <.10
